# Analysis of the expression of the Notch3 receptor protein in adult lung cancer

**DOI:** 10.3892/ol.2012.1033

**Published:** 2012-11-19

**Authors:** MIN ZHOU, WEI-YUN JIN, ZHI-WEN FAN, RUI-CHAO HAN

**Affiliations:** 1 Department of Respiratory Medicine, Jinshan Branch of the Sixth People’s Hospital of Shanghai, Shanghai Jiao Tong University, Shanghai 201500;; 2 Department of Respirtory Medicine, The Third People’s Hospital Affiliated to Shanghai Jiao Tong University School of Medicine, Shanghai 201999, P.R. China

**Keywords:** bronchiogenic carcinoma, Notch3, lung cancer

## Abstract

Abnormalities in the Notch signaling system are considered to play a role in the tumorigenesis of bronchiogenic carcinoma. The present study aimed to investigate the expression of Notch3 in adult lung cancer patients and its role in the pathogenesis of primary bronchiogenic carcinoma. The expression of the Notch3 protein in lung squamous cell carcinoma, adenocarcinoma, small cell carcinoma and corresponding non-tumor tissues was detected by immunohistochemistry. To investigate the expression of Notch3 in adenocarcinoma tissues, Notch3 mRNA and protein expression were measured with reverse transcription polymerase chain reaction (RT-PCR) and western blot analysis, respectively. It was demonstrated that Notch3 had a stronger positive degree of expression in lung squamous cell carcinoma and adenocarcinoma compared with the corresponding non-tumor tissue (P<0.01). The expression of Notch3 in small cell carcinoma tissue was lower compared with that of the corresponding non-tumor tissue (P<0.01). The expression of Notch3 in the lung adenocarcinoma group was the highest of the three lung carcinoma groups (P<0.01). RT-PCR revealed that the expression of Notch3 mRNA in the lung adenocarcinoma group was higher than that of the normal lung group, but there was no statistically significant difference (P=0.05). The expression of Notch1 protein in the lung adenocarcinoma group was significantly higher compared with the normal lung group (P<0.01), as shown by western blot analysis. Notch3 may be involved in the pathogenesis of bronchogenic carcinoma, in particular in the promotion of the lung cancer oncogene, and a difference in its expression may exist in the various pathological types.

## Introduction

Primary bronchial lung cancer (referred to as lung cancer) is the most common primary pulmonary malignant tumor, despite progress in surgical techniques, chemotherapy and radiotherapy. The 5-year survival rate of patients with lung cancer remains very low. Jemal *et al* revealed that the 5-year survival rate of patients with lung cancer was only 16% (1). It is currently thought that abnormality of the multi-intracellular transmission pathway is associated with lung cancer, and there is increasing interest in the role of Notch signaling systems in tumorigenesis (2). Four notch genes encoding four types of Notch receptors, Notch1–4, have been identified in mammals. At least five types of ligand are capable of binding with these receptors and altering their function. Previously, studies concerned with the pathogenesis of lung cancer have identified a differential expression of Notch components in different types of lung cancer. It is hypothesized that Notch1 and 3 are associated with non-small cell lung cancer (NSCLC) (3,4). The majority of studies consider that Notch1 is involved in cancer promotion (5,6) and, compared with Notch1, there are a small number of studies regarding Notch3. Additionally, whether the effects of Notch1 and 3 are similar remains controversial.

To further investigate the role of Notch signaling in lung adenocarcinoma, on the basis of Notch1 research, cases of surgical resection of small cell lung cancer (SCLC), lung squamous cell carcinoma and adenocarcinoma for the past 2 years in our hospital were reviewed. Immunohistochemistry and imaging analysis were used to detect the expression of Notch3 in lung cancer and negative tissue margins. Preliminary results indicated that the differences in Notch3 expression in lung adenocarcinoma were the most prominent and, accordingly, those specimens were collected from adult thoracic (lung) cancer surgerical resections. Specimens were later diagnosed as lung adenocarcinoma in post-operative pathological sections or normal lung tissue samples between January 2011 and May 2012. The expression of Notch3 in lung cancer tissues was detected by reverse transcription polymerase chain reaction (RT-PCR) and western blot analysis to further explore the role of Notch3 in adult lung adenocarcinoma.

## Materials and methods

### Study population and tissue samples

Fify-seven archived lung cancer wax blocks were collected from thoracic surgical resections between January 2010 and December 2011 at the Jinshan Branch of the Sixth People’s Hospital of Shanghai, Shanghai Jiao Tong University, Shanghai, China. Of the 55 blocks, 20 cases were squamous cell carcinoma, 24 were adenocarcinoma and 13 were small cell carcinoma. The general clinical data of these patients are shown in Table I. One cancerous tissue embedded in a paraffin wax block was selected with the corresponding non-tumor tissue of the wax block. Three groups of non-tumor tissue in the wax block specimens were missing (partially absent). Additionally, serial sections (4 μm) were cut for analysis.

The specimens of adult lung cancer from thoracic surgery, which were later diagnosed by post-operative pathological section analysis as lung adenocarcinoma, were studied as the experimental group, and the specimens of the corresponding non-tumor tissues were studied as the control group. There were 13 lung adenocarcinoma patients, comprising 9 males and 4 females, aged 40–72 years (mean, 59.5 years).

The study was conducted according to the principles of the Declaration of Helsinki. Informed consent was obtained and the Ethics Committee of Jinshan Branch of the Sixth People’s Hospital, Shanghai Jiao Tong University approved the study. The patient data, which are contained within this article, were obtained by a hospital-based doctor at Jinshan Branch of the Sixth People’s Hospital, Shanghai Jiao Tong University. Permission to use these data in this report has been obtained from all the subjects who participated in this study (7).

### Hematoxylin and eosin (H&E) staining and immunohistochemistry

Prepared paraffin sections from each group were stained conventionally with H&E to determine whether there was consistency with the pathological type determined in the original study.

The aforementioned prepared paraffin sections were studied according to standard procedures. The working concentrations of Notch3 antibody (Cell Signaling Technology, Inc., Danvers, MA, USA) and glyceraldehyde 3-phosphate dehydrogenase (GAPDH; Shanghai Kangcheng Biotechnology Co., Ltd., Shanghai, China) were 1:200 and that of HRP-marked secondary antibody (Shanghai Weiao Biotech Ltd., Shanghai, China) was 1:100. Phosphate-buffered saline (PBS) was selected as the negative control as opposed to the primary antibody.

### Evaluation of Notch3 immunostaining

Cytoplasm and/or nuclei with brown particles were declared as positive. The imaging analysis was performed by an immunohistochemical slice set under an Olympus (Tokyo, Japan) microscope (×200); the accurate position of the measured visual field was determined and then three complete visual fields without overlap were randomly selected for viewing. Image-Pro Plus 6.0 software was used to analyze the area, the integral optical density (IOD), the number of positive cells and the total cell number in each visual field. The mean IOD of each slice was set as the measured value for the case.

### Surgical specimens and HE staining

Fresh specimens obtained via surgical resection were sent to the Department of Pathology at the Jinshan Branch of the Sixth People’s Hospital of Shanghai, Shanghai Jiao Tong University, Shanghai, China. Under the guidance and assistance of the pathologist, the samples (two samples of lung cancer tissue and two samples of normal control lung tissue) were immediately drawn, placed into Eppendorf (EP) tubes (1.5 ml, sterilized and enzyme inactivated) and stored at −80°C. Another sample from the same position as the cancerous tissue and a normal control lung tissue sample were drawn and fixed in 10% neutral formalin for >24 h for H&E staining.

### Semiquantitative RT-PCR

RNA extraction was carried out on the samples of cancerous and normal control lung tissue with TRIzol reagent. The RNA concentration and purity were detected with a spectrophotometer. RT-PCR was performed with a Takara RT-PCR kit according to the manufacturer’s instructions (Takara Bio., Inc., Shiga, Japan). The amplified products of Notch3 and GAPDH were 419 and 309 bp, respectively. The primer sequences were as follows: Upstream, AATGCCAACTGAAGAGGATGAG and downstream, AGTGTAAGGCTGATTTCCCAAG for Notch3; upstream, TCCCATCACCATCTTCCAG and downstream, ATGAGTCCTTCCACGATACC for GAPDH. PCR was performed in a 25-μl reaction mixture. The conditions for Notch3 and GAPDH PCR were one cycle of denaturing at 94°C for 2 min, followed by 40 (Notch3) or 25 (GAPDH) cycles at 94°C for 30 sec, and at 60°C (Notch3) or 55°C (GAPDH) for 30 sec, prior to a final extension at 72°C for 1 min.

Following agarose gel electrophoresis (80 V, 60 min), the electrophoresed PCR products were scanned by densitometry. The relative value of the Notch3 band to the GAPDH band was calculated in each sample.

### Western blot analysis

The samples of cancerous tissue and normal control lung tissue were lysed in a protein lysate. After the cellular protein was extracted, equal amounts (40 μg) of protein were resolved on precast gels (Gradipore Ltd., Frenchs Forest, Australia) and transferred to polyvinylidene fluoride (PVDF) membranes. The blots were probed with 1:200 Notch3 monoclonal antibody (Cell Signaling Technology, Inc.), GAPDH monoclonal antibody (Shanghai Kangcheng Biotechnology Co., Ltd.) and hoseradish peroxidase (HRP)-marked secondary antibody (Shanghai Weiao Biotech Ltd.). The bound antibodies were visualized with a Photope-HRP Western Detection kit (Cell Signaling Technology, Inc.). The relative value of the Notch3 band to the GAPDH band was calculated in each sample with Quality-one software.

### Statistical analysis

Statistical analyses were performed using the Statistical Package for the Social Sciences (SPSS) 17.0 software (SPSS Inc., Chicago, IL, USA). A one-way analysis of variance (ANOVA) was used to test for significant differences in measured variables between groups. P<0.05 was considered to indicate a statistically significant difference.

## Results

### Pathomorphology of paraffin sections of each group and immunohistochemical analysis for Notch3 protein

**Figure 1.**
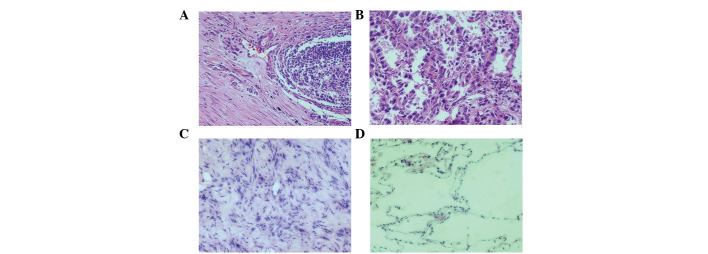
Hematoxylin and eosin (H&E) staining of subtypes of cancerous tissue and their corresponding non-tumor tissue (×200). (A) Lung squamous cell carcinoma: Cancer cells are polygonal with a greater amount of cytoplasm, deep nuclear staining and intercellular bridges. Keratin pearls are shown. (B) Lung adenocarcinoma: Glandular structures are visible in the cancer tissue and cancer cells are comparatively large with abundant cytoplasm and large nuclei containing secretory granules or mucocysts. (C) Small cell lung cancer: Cancer cell morphology is similar to the oat grain with relative consistency in size, a large nucleus and deep staining. (D) Non-tumor tissue: The structure is similar to that of normal lung tissue and certain specimens exhibit inflammatory cell infiltration.

The selected specimens were confirmed to be consistent with the lung cancer sub-types following observation with H&E staining (Fig. 1).

**Figure 2.**
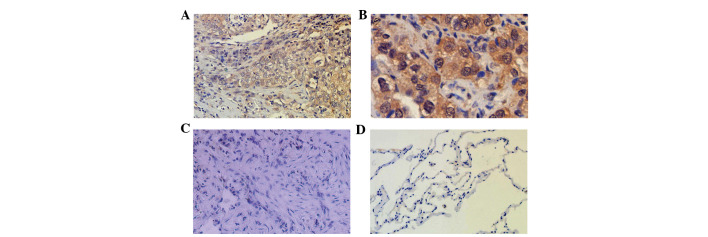
Notch3 expression in subtypes of cancerous tissue and corresponding non-tumor tissue (IHC, ×200). (A) Lung squamous cell carcinoma: Positive staining is evident in the cytoplasm and nucleus. (B) Lung adenocarcinoma: Stronger positive staining is visible in the cytoplasm and nucleus. (C) Small cell lung cancer: Positive staining is not evident in the cancer cells. (D) Non-tumor tissue. Positive staining is shown in the small vessels and weaker staining is observed in the alveolar epithelial cells in non-tumor tissues.

By immunohistochemisty, positive Notch3 expression was observed in each group in the vascular smooth muscle of small vessels, while weaker staining was observed in the alveolar epithelial cells of non-tumor tissues. Positive Notch3 expression was identified in the cytoplasm and nuclei in the squamous cell carcinoma and adenocarcinoma groups, and the adenocarcinoma groups demonstrated a stronger positive expression. However, the positive staining was not clear in the SCLC group. Following the statistical analysis of Notch3-positive cellular responses, the results demonstrated that there were no significant differences in the non-tumor tissue of the squamous cell carcinoma, adenocarcinoma and small cell carcinoma groups (P>0.05). Notch3 expression in the cancer tissue was stronger than that of the corresponding non-tumor tissue in the squamous cell carcinoma and adenocarcinoma groups; the difference was statistically significant (P<0.01). Notch3 expression in the cancer tissue was weaker than that of the corresponding non-tumor tissue in the SCLC group; the difference was statistically significant (P<0.01). The highest Notch3 expression was observed in the adenocarcinoma group compared with the other two groups and the difference was statistically significant (P<0.01; Fig. 2 and Table II).

### Semiquantitative RT-PCR analysis of Notch3 mRNA

**Figure 3.**
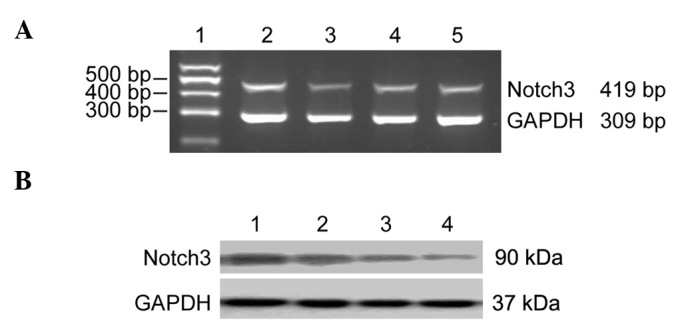
Expression of Notch3 mRNA and protein in lung adenocarcinoma and normal lung tissue. (A) Notch3 mRNA levels as analyzed by RT-PCR: Lung adenocarcinoma tissue (lanes 2 and 4) expressed higher levels of Notch3 mRNA compared with normal lung tissue (lanes 3 and 5). The integral optical density (IOD) value statistical analysis revealed no significant difference in mRNA expression (P>0.05). (B) Notch3 protein levels as analyzed by western blot analysis in lung adenocarcinoma and normal lung tissue. Lung adenocarcinoma tissue (lanes 1 and 2) expressed higher levels of Notch3 protein compared with normal lung tissue (lanes 3 and 4). IOD values revealed a significant difference in protein expression (P<0.01).

The observation of pathological sections with H&E staining of selected lung tissue confirmed the specimens were adenocarcinoma and paraneoplastic normal lung tissues, in accordance with the findings of the Department of Pathology. There were 13 adult lung adenocarcinomas and 13 paraneoplastic normal lung tissue specimen cases. According to the RT-PCR results, the expression of Notch3 mRNA in the lung adenocarcinoma group was higher than that of the paraneoplastic normal lung tissues; however, statistical analysis showed that the integrated optical density (IOD) values were not significantly different between the 2 groups (P>0.05; Fig. 3 and Table III).

### Expression of Notch3 protein in lung adenocarcinoma

According to the western blot analysis results, the expression of Notch3 protein in the lung adenocarcinoma group was higher than that of the paraneoplastic normal lung tissue, and the statistical analysis demonstrated that the integrated optical density (IOD) values were not significantly different (P<0.01; Fig. 3 and Table IV).

## Discussion

Notch is a highly evolutionarily conserved single transmembrane receptor protein that plays an important role in deciding cell fate. It is a key regulatory factor in cell differentiation, proliferation and apoptosis. Four types of Notch genes have been found in mammals coding four Notch receptors (Notch1–4). At least 5 types of ligand bind to Notch: Jagged1 and 2 and Delta1, 3 and 4. HES/HEY family members are genes that act downstream of Notch, and have different gene distributions in different tissues. One study regarding the integrative genomic analyses of the HES/HEY family presumed that Notch-independent HES1 and 3 transcription occurred in undifferentiated ES cells, and that Notch-dependent HES1 and 5, HEY1 and 2, and HEYL transcription occurred in fetal, adult or cancer tissue (8). Notch signals are closely related to certain regulatory factors in multiple tumorigenesis. The Notch ligand, JAG1, which functions as a WNT-dependent Notch signaling activator, is the key molecule maintaining homeostasis of stem and progenitor cells (9). γ-secretase cleaving Notch into the Notch intercellular domain (NICD) is the key step in the conduction of Notch signals. One study investigating tumor angiogenesis suggested that administration of the γ-secretase inhibitor may be combined with disruption of eNOS or interruption of VEGF signaling (10). This phenomenon may be explained by the upstream regulation of Notch (11). Notch signals are located upstream of signal transduction for other regulatory factors associated with tumorigenesis, including NFAT, NF-κB, T-Bet and GATA3 (12). Therefore, the role of Notch in the occurrence and development of certain tumors in lung cancer has been confirmed. Abnormal expression of Notch1 was first identified in human T lymphocytes in acute lymphoblastic leukemia (13). Subsequent studies have confirmed that Notch subtypes are differentially expressed and have various roles in different types of tumors (14–17).

There has been an increasing interest in Notch in lung cancer research. Primary bronchogenic carcinoma of the lung may be summarized as either SCLC or NSCLC, and NSCLC mainly includes lung squamous cell carcinoma and adenocarcinoma. At present, the role of Notch in lung carcinogenesis has not been determined. The Notch signaling pathway either promotes or inhibits lung cancer, and it is inferred that this may be related to a number of factors, such as different types of tumor cells, subtypes of Notch and ligands (4,18). Notch1 and 3 are considered to have a relatively close correlation with lung cancer (3,4). Notchl signal activation is considered to promote the growth of tumor cells of NSCLC and inhibit the growth of SCLC (19,20). Studies on Notch3, compared with Notch 1, remain small in number, and whether Notch3 and Notch1 have a syntropic effect is unknown. In our preliminary studies, the expression of Notch1 in lung squamous cell carcinoma, adenocarcinoma and SCLC specimens was detected by immunohistochemical methods. The experimental results demonstrated a high Notch1 expression in lung adenocarcinoma and squamous cell carcinoma, while low Notch1 expression was observed in SCLC. Differences also exist in the two types of high expression (21,22). The expression of Notch1 and 3 was detected in the lung tissue of asthma model mice and they demonstrated reverse changes (23).

To discover the role of Notch3 in lung cancer and its correlation with Notch1, by reviewing the specimens of SCLC, lung squamous cell carcinoma and adenocarcinoma removed during surgery in the last two years, the expression of Notch3 in each group of lung cancer tissue and its corresponding non-tumor tissue was detected by an immunohistochemical method on the basis of the study of Notch1. The results revealed that among the three groups of specimens of lung cancer, the expression of Notch3 was the highest in squamous cell carcinoma and adenocarcinoma, and was the lowest in SCLC. The difference between the groups was statistically significant. Among the three groups of lung cancer specimens, the expression of Notch3 in lung adenocarcinoma was the highest. Accordingly, we collected specimens of adult lung cancer during thoracic surgery, which were later diagnosed as lung adenocarcinoma and normal lung tissue, between January 2011 and May 2012 at the Jinshan Branch of the Sixth People’s Hospital of Shanghai, Shanghai Jiao Tong University, Shanghai, China. The expression of Notch3 in the lung cancer tissue was detected by RT-PCR and western blot analysis. The results demonstrated that the expression of Notch3 protein in the lung adenocarcinoma group was higher than that of the normal paraneoplastic lung tissues. Additionally, the expression of Notch3 mRNA in the lung adenocarcinoma group was higher than that of the normal paraneoplastic lung tissues. However, the IOD values showed no significant difference between the two groups. The reasons for the inconsistency between the levels of nucleic acid and protein expression, as analyzed, may be associated with the small number of experimental specimens and less precise experimental methods. This type of error may be corrected by applying a real-time PCR method. It may be inferred from the experimental results that Notch3 has a certain type of effect, which varies in the different pathological types of lung cancer and shows a syntropic effect with Notch1. However, whether there is an additive effect requires further study. There are few studies on other Notch subtypes in lung cancer. Clarification of the correlation between Notch signaling and primary lung cancer incidence has great significance.

## Figures and Tables

**Table I.** General clinical data of 57 lung cancer cases.

	A (squamous cell carcinoma)	A1 (negative margin)	B (adenocarcinoma)	B1 (negative margin)	C (small cell carcinoma)	C1 (negative margin)
Case number	20	18	24	21	13	11
Mean age (years)	68.9		63.5		64.9	
Gender ratio (male:female)	17:3		18:6		10:3	

**Table II.** Notch3 expression in subtypes (mean ± standard deviation).

Group	Number of cases	Integrated optical density
A^a,d^	20	5920.56±928.60
A1	18	1693.64±439.25
B^b,c^	24	20074.32±1264.08
B1	21	1740.38±360.32
C^d^	13	265.09±78.31
C1	11	1864.21±373.63

A, squamous cell carcinoma group; A1, corresponding non-tumor tissue group to the squamous cell carcinoma group; B, adenocarcinoma group; B1, corresponding non-tumor tissue group to the adenocarcinoma group; C, small cell carcinoma group; C1, corresponding non-tumor tissue group to the small cell carcinoma group.

a P<0.01 vs. group A1;

b P<0.01 vs. group B1;

c P<0.01 vs. groups A and C;

d P <0.01 vs. group C.

**Table III.** Integral optical density values of Notch3 mRNA in lung adenocarcinoma tissue (mean ± standard deviation, n=13).

Group	Notch3 mRNA	P-value
Lung adenocarcinoma group	0.32±0.05	
Non-tumor tissue group	0.28±0.06	0.05

**Table IV.** Integral optical density values of Notch3 protein in lung adenocarcinoma tissue (mean ± standard deviation, n=13).

Group	Notch3 protein	P-value
Lung adenocarcinoma group	0.28±0.06	
Paraneoplastic group	0.19±0.03	<0.01
